# Myalgic Encephalomyelitis—Chronic Fatigue Syndrome Common Data Element item content analysis

**DOI:** 10.1371/journal.pone.0291364

**Published:** 2023-09-12

**Authors:** Mary D. Slavin, Hannah M. Bailey, Emily J. Hickey, Ananya Vasudevan, Aileen Ledingham, Linda Tannenbaum, Lucinda Bateman, David L. Kaufman, Daniel L. Peterson, Ilene S. Ruhoy, David M. Systrom, Donna Felsenstein, Lewis E. Kazis

**Affiliations:** 1 Department of Health Law Policy and Management, Boston University School of Public Health, Boston, Massachusetts, United States of America; 2 Spaulding Rehabilitation Hospital, Rehabilitation Outcomes Center (ROC), Boston, Massachusetts, United States of America; 3 University Center for Excellence in Developmental Disabilities, Waisman Center, University of Wisconsin-Madison, Madison, Wisconsin, United States of America; 4 Boston University School of Medicine, Boston, Massachusetts, United States of America; 5 Open Medicine Foundation, Agoura Hills, California, United States of America; 6 Bateman Horne Center of Excellence, Salt Lake City, Utah, United States of America; 7 Center for Complex Diseases, Mountain View, California, United States of America; 8 Sierra Internal Medicine, Incline Village, Nevada, United States of America; 9 Mount Sinai South Nassau, Neurology, Chiari/EDS Center, Oceanside, New York, United States of America; 10 Brigham and Women’s Hospital, Lung Center, Boston, Massachusetts, United States of America; 11 Massachusetts General Hospital, Boston, Massachusetts, United States of America; 12 Harvard Medical School, Boston, Massachusetts, United States of America; IRCCS Medea: Istituto di Ricovero e Cura a Carattere Scientifico Eugenio Medea, ITALY

## Abstract

**Introduction:**

Myalgic Encephalomyelitis/Chronic Fatigue Syndrome (ME/CFS) is a multisystem chronic disease estimated to affect 836,000–2.5 million individuals in the United States. Persons with ME/CFS have a substantial reduction in their ability to engage in pre-illness levels of activity. Multiple symptoms include profound fatigue, post-exertional malaise, unrefreshing sleep, cognitive impairment, orthostatic intolerance, pain, and other symptoms persisting for more than 6 months. Diagnosis is challenging due to fluctuating and complex symptoms. ME/CFS Common Data Elements (CDEs) were identified in the National Institutes of Health (NIH) National Institute of Neurological Disorders and Stroke (NINDS) Common Data Element Repository. This study reviewed ME/CFS CDEs item content.

**Methods:**

Inclusion criteria for CDEs (measures recommended for ME/CFS) analysis: 1) assesses symptoms; 2) developed for adults; 3) appropriate for patient reported outcome measure (PROM); 4) does not use visual or pictographic responses. Team members independently reviewed CDEs item content using the World Health Organization International Classification of Functioning, Disability and Health (ICF) framework to link meaningful concepts.

**Results:**

119 ME/CFS CDEs (measures) were reviewed and 38 met inclusion criteria, yielding 944 items linked to 1503 ICF meaningful concepts. Most concepts linked to ICF Body Functions component (b-codes; n = 1107, 73.65%) as follows: Fatiguability (n = 220, 14.64%), Energy Level (n = 166, 11.04%), Sleep Functions (n = 137, 9.12%), Emotional Functions (n = 131, 8.72%) and Pain (n = 120, 7.98%). Activities and Participation concepts (d codes) accounted for a smaller percentage of codes (n = 385, 25.62%). Most d codes were linked to the Mobility category (n = 69, 4.59%) and few items linked to Environmental Factors (e codes; n = 11, 0.73%).

**Discussion:**

Relatively few items assess the impact of ME/CFS symptoms on Activities and Participation. Findings support development of ME/CFS-specific PROMs, including items that assess activity limitations and participation restrictions. Development of psychometrically-sound, symptom-based item banks administered as computerized adaptive tests can provide robust assessments to assist primary care providers in the diagnosis and care of patients with ME/CFS.

## Introduction

Myalgic Encephalomyelitis/Chronic Fatigue Syndrome (ME/CFS) is an acquired, chronic complex condition. While the etiology is uncertain, ME/CFS is often preceded by an acute viral infection [1–7] and has been associated with neurological, metabolic, immunological and autonomic nervous system dysfunction. It is estimated that 836,000 to 2.5 million people in the United States have ME/CFS, although the actual number may be higher due to under-diagnosis [8]. ME/CFS is a serious, life-changing condition. Up to 75% of persons with ME/CFS either cannot work or require a decreased work schedule; 25% are consistently home- or bed-bound [9–11]; and few persons return to their level of pre-illness health [12, 13].

Patients with ME/CFS present with the following symptoms: extreme fatigue; cognitive impairments, often described as brain fog; unrefreshing sleep; autonomic dysfunction, including orthostatic intolerance; sensory sensitivities; gastrointestinal problems; chemical sensitivity; muscle and/or joint pain; headaches; and flu-like symptoms [1]. The hallmark symptom of ME/CFS is post-exertional malaise (PEM) [14, 15], which presents as a prolonged worsening of symptoms and further reduction of cognitive and/or physical functioning after engaging in previously tolerated activities [16]. The National Academy of Medicine (NAM) ME/CFS diagnostic criteria require the presence of three key symptoms: 1) a substantial reduction in activity persisting for more than 6 months and accompanied by profound fatigue; 2) PEM; and 3) unrefreshing sleep. At least one of the following symptoms must also be present: cognitive impairment or orthostatic intolerance. Symptoms need to be present at least half of the time with moderate, substantial, or severe intensity for more than six months [1].

Due to the complexity of symptom presentation, screening patients for ME/CFS is often challenging. Given the severe impact of ME/CFS and the difficulty in obtaining a diagnosis, there is an urgent need for better tools to assist in identifying and managing the care of persons with ME/CFS. Efforts to identify ME/CFS assessments for use in research studies culminated in development of Common Data Elements (CDEs) [17]. ME/CFS CDEs include 119 measures that assess a range of symptoms [18, 19], providing the most comprehensive published list of assessments currently available.

**Fig 1:**
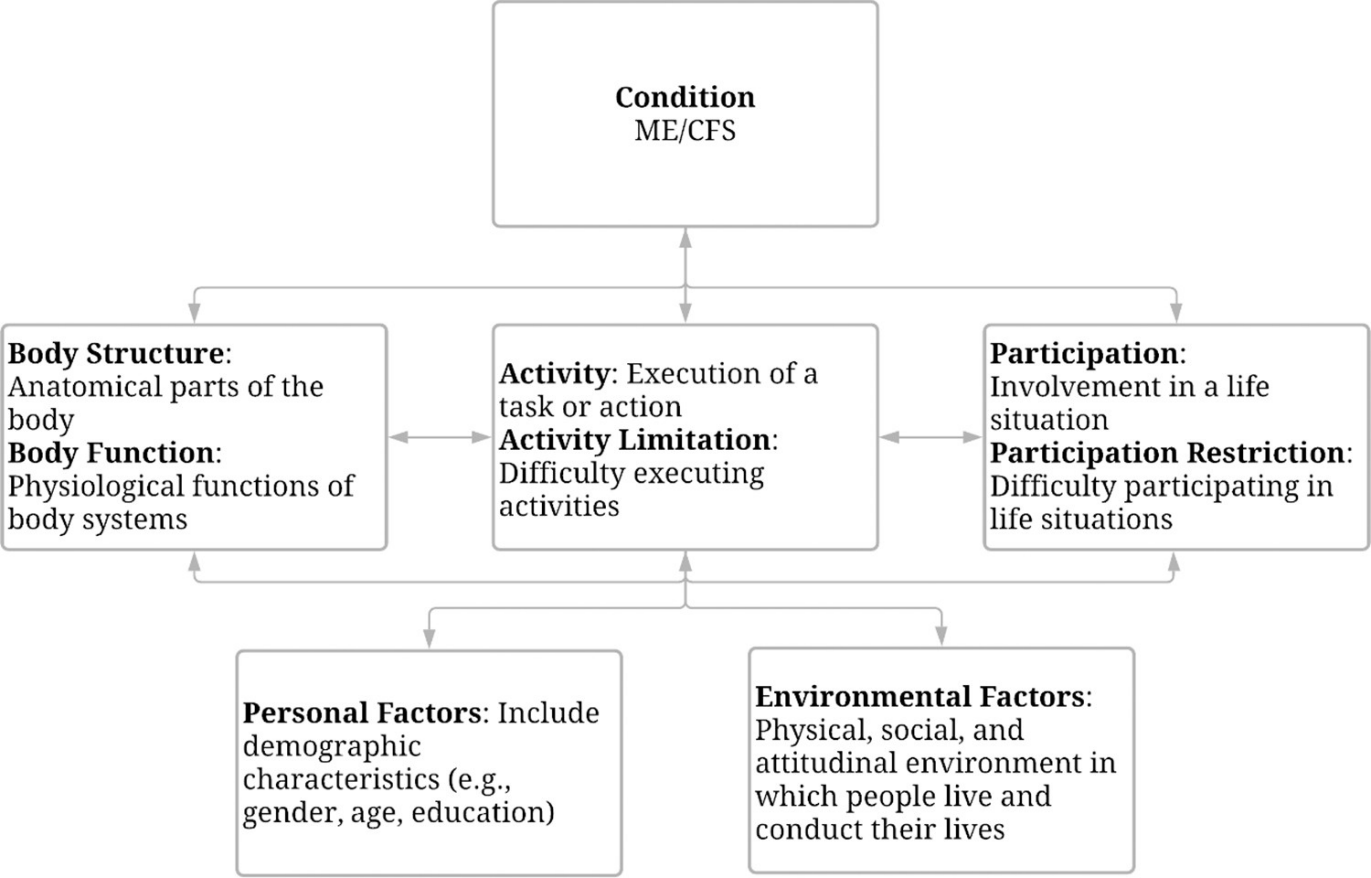
International Classification of Functioning, Disability and Health (ICF) applied to ME/CFS.

The large number of ME/CFS CDEs and complexity of ME/CFS symptoms contribute to challenges in optimizing assessment selection. Applying an established conceptual framework to analyze CDEs content can serve as a guide for selecting CDEs. The World Health Organization (WHO) International Classification of Functioning, Disability and Health (ICF) is a well-established conceptual framework, and it is widely used to classify health and health-related domains. The ICF has been extensively researched and validated over the last two decades [20–22]. Fig 1 illustrates the ICF framework as applied to ME/CFS. The ICF framework includes five major components: Body Functions *(physiological and psychological functions of body systems)*; Body Structures *(anatomical parts of the body*, *such as organs*, *and limbs)*; Activities *(execution of tasks at an individual level* and Participation (*the individual’s involvement in everyday life situations)*; Environmental Factors *(physical*, *social*, *and attitudinal factors in the person’s life and society which hinder or facilitate the functioning of the individual)*, and Personal Factors *(characteristics unique to individuals such as age*, *gender*, *ethnicity*, *personality*, *resilience*, *or experiences* [23].

The overall purpose of the study is to understand the relative strengths and limitations of ME/CFS CDEs. The study reviewed item content based on the ICF conceptual framework to accomplish the following aims: classify item content and analyze item content coverage [24, 25]. Study findings will serve as a guide to optimize selection of ME/CFS CDEs and inform future efforts to develop new assessments.

## Methods

First, ME/CFS CDEs were reviewed and selected based on inclusion criteria. ME/CFS CDEs include a range of clinical assessments and patient-reported outcome measures (PROMs). This analysis focused on ME/CFS CDEs appropriate for administration as PROMs. PROMs provide insights derived directly from patient experiences [26] and are widely recognized as valuable tools to assess changes in health status and the impact of health conditions on quality of life [27]. Additional study inclusion criteria were: 1) assesses symptoms; 2) used in adults; and 3) does not use visual or pictographic responses. Four members of the research team (MDS, HMB, AV, EH) reviewed the 119 ME/CFS CDEs and identified assessments that met inclusion criteria.

### Item content classification

Items from the included assessments were reviewed to describe and classify item content based on the ICF framework. Previous work has established the ICF core sets for patients with ME/CFS [28] and a systematic review of PROMs used in ME/CFS has been completed [29]. Methods to link assessment items to the ICF are well established and are commonly used in measurement development to ensure that items assess relevant content [30–32]. ICF item linking methods include two steps: 1) review each item to identify meaningful concept(s); 2) select the most precise ICF category for each meaningful concept(s) [24, 25].

Items from the 119 ME/CFS CDEs were entered into an Excel spreadsheet to track item content analyses. Each item was randomly assigned to two team members for independent review. First, team members identified meaningful concept(s) and reached agreement if discrepancies were noted. Next, two team members reviewed meaningful concept(s) and selected the ICF category/subcategory that best matched the concept. Any disagreement was brought to the larger group for discussion until consensus was reached. The ICF defines categories and subcategories by using hierarchically organized alphanumeric codes. Letters define the component (b = Body Function, d = Activity and Participation, e = Environment) and numbers describe up to four categories/subcategories. For example, the ICF code for the Quality of Sleep is b1343: b (body function), 134 (sleep functions), 3 (quality of sleep). The team entered the ICF code linked to each concept into the spreadsheet for content analyses.

### Item content linking analyses

The following analyses were conducted: 1) determine the distribution of meaningful concepts in CDE items across ICF Components (Component codes divided by total ICF codes); 2) analyze the distribution of CDE item meaningful concepts based on ME/CFS symptoms (ICF categories/subcategories codes divided by total codes); 3) examine item content (ICF categories/subcategories codes divided by total items); 4) analyze content for the three most commonly used ME/CFS CDEs.

## Results

**Fig 2:**
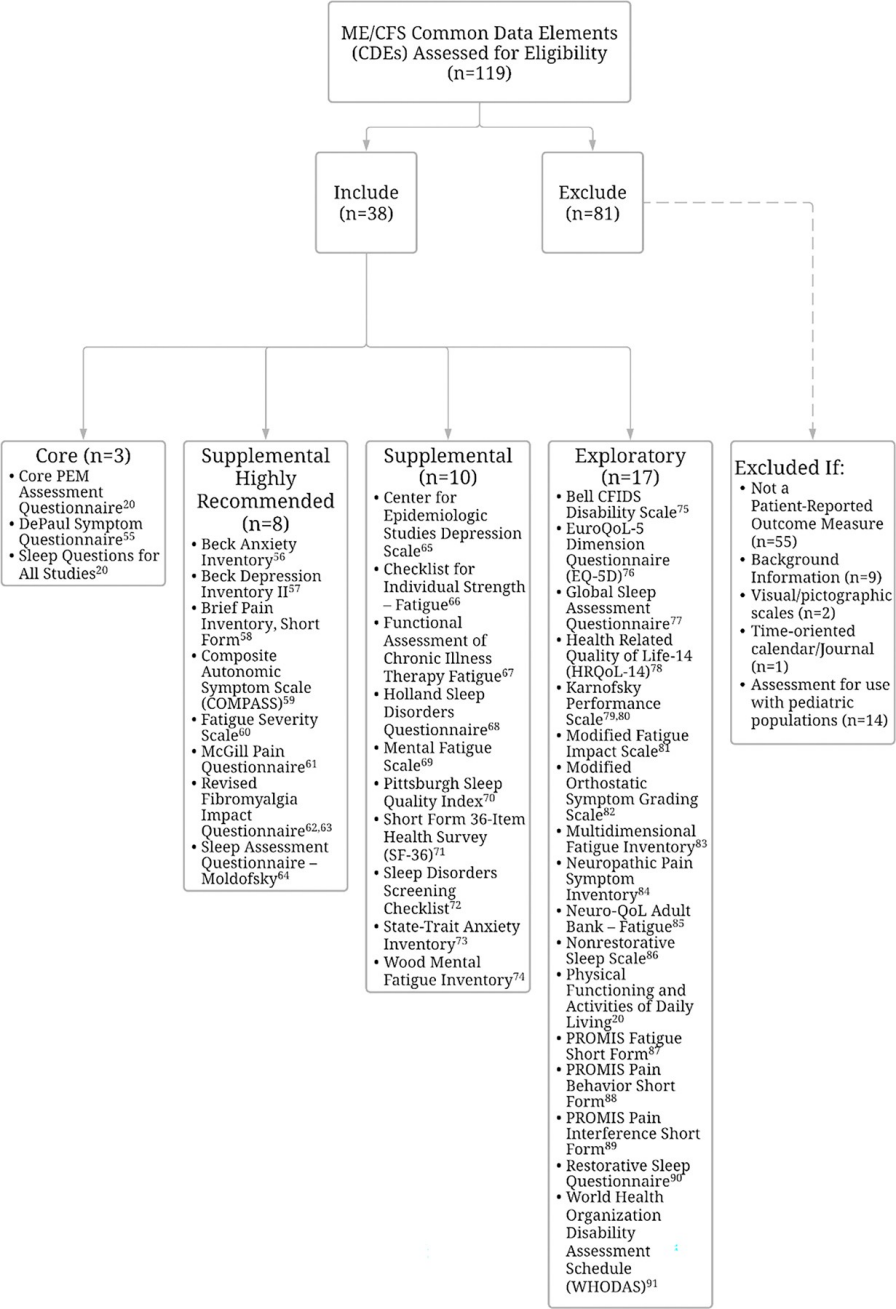
NIH ME/CFS Common Data Elements, inclusion and exclusion.

Fig 2 summarizes the ME/CFS CDEs selection review process. Of the 119 ME/CFS CDEs reviewed, 38 met inclusion criteria. Excluded assessments are summarized in S1 Appendix. The 38 assessments were comprised of 944 items that were reviewed and analyzed. In total, 1584 meaningful concepts were identified. Some meaningful concepts (n = 81, 5.39%) were categorized as “non-definable,” “not covered,” and “health condition” and these concepts were omitted from the content analysis summary. The remaining 1503 meaningful concepts were linked to an ICF category and subcategory. Nearly half of 944 items reviewed included meaningful concepts linked to more than one ICF category (n = 427, 45.23%). No meaningful concepts were linked to the ICF Body Structure Component.

### ICF content linking analyses: Body functions component

Table 1 summarizes the distribution of ME/CFS CDEs meaningful concepts (n = 1503) across ICF Body Functions codes. The majority of meaningful concepts were coded as ICF Body Functions component (n = 1107, 73.65%). The highest representation of ICF Body Function codes were related to the ME/CFS symptom of fatigue (n = 433, 28.81%), which is identified as a required symptom according to the NAM diagnostic criteria (1). Meaningful concepts that reflected a physical function component of fatigue were linked to Fatiguability, defined as “Functions related to susceptibility to fatigue, at any level of exertion” (ICF code b4552; n = 220, 14.64%). Meaningful concepts that reflected a mental function component of fatigue were linked to Energy Level, defined as “mental functions that produce vigor and stamina” (ICF code b1300; n = 166, 11.04%). Meaningful concepts related to fulfilling needs were linked to Energy and Drive Functions, defined as mental functions that “cause the individual to move towards satisfying specific needs and general goals in a persistent manner” (ICF code b130; n = 47, 3.13%). If the meaningful concept was an unspecified lack of energy or it was unclear if the concept was related to the physical or mental component, both ICF codes were assigned.

**Table 1:** ICF content linking analysis: Body functions component.

Symptom	Categories and Subcategories	Code Count	Code Percent*
Fatigue	Fatiguability (b4552)	n = 220	14.64
	Energy level (b1300)	n = 166	11.04
	Energy and drive functions (b130)	n = 47	3.13
**Fatigue Total**		**n = 433**	**28.81**
Unrefreshing Sleep	b134 (Sleep Functions), 1340 (Amount of sleep), 1341 (Onset of sleep), 1342 (Maintenance of sleep), 1343 (Quality of sleep), 1344 (Functions involving the sleep cycle)	n = 137	9.12
Cognitive function	b140 (Attention functions), 1400 (Sustaining attention), 1402 (Dividing attention), 144 (Memory functions), 1440 (Short-term memory), 1442 (Retrieval and processing of memory), 1443 (Working memory),160 (Thought functions), 1600 (Pace of thought), 164 (Higher-level cognitive functions), 1641 (Organization and planning), 1644 (Insight), 1646 (Problem solving)	n = 79	5.26
Orthostatic Intolerance	b2401 (Dizziness), 2402 (Sensation of falling), 4100 (Heart rate), 4101 (Heart rhythm), 415 (Blood vessel functions)	n = 14	0.93
Emotional functions**	b152 (Emotional functions), 1520 (Appropriateness of emotion), 1522 (Range of emotion)	n = 131	8.72
Pain**	b280 (Sensation of pain), 2800 (Generalized pain), 2801 (Pain in body part), 28010 (Pain in head and neck), 28011 (Pain in chest), 28012 (Pain in stomach or abdomen), 28016 (Pain in joints)	n = 120	7.98
Fewer than 20 codes		n = 193	12.84
TOTAL		n = 1107	73.65

*Based on Total Codes (N = 1503)

**Symptom not included in the NAM diagnostic framework

Additional ICF Body Function categories/subcategories were related to other ME/CFS symptoms, as defined by the NAM diagnostic criteria (1). Unrefreshing sleep included six ICF subcategories (n = 137, 9.12%); cognitive function included 13 ICF subcategories (n = 79, 5.26%); and orthostatic intolerance included five ICF subcategories (n = 14, 0.93%). Other ICF Body Function categories/subcategories assessed content related to symptoms not described in the NAM criteria (1) including content related to emotional functions (n = 131, 8.72%) and seven subcategories describing pain (n = 120, 7.98%). ICF categories with fewer than 20 codes were not detailed for presentation (n = 193, 12.84%).

### ICF content linking analysis: Activities and participation component

Compared to Body Functions, a smaller percent of meaningful concepts were linked to the ICF Activities and Participation component (n = 385, 25.62%). Table 2 presents Activities and Participation content analysis by chapter and category/subcategory. The Mobility chapter had the highest representation of ICF concepts (n = 69, 4.59%) with 20 categories and subcategories describing different aspects of Mobility (e.g., moving around within the house, changing and maintaining body position). Content related to the Self-Care chapter (n = 49, 3.26%) had the next highest representation and included nine categories and subcategories describing different aspects of Self-Care (e.g., washing oneself, looking after one’s health). Several items were linked to Remunerative Employment (n = 39, 2.59%). The General Tasks and Demands (n = 30, 2.00%) category was linked to general aspects of carrying out single or multiple tasks, organizing routines and handling stress. Carrying out Daily Routine (n = 37, 2.46%) was linked to items that assessed simple or complex and coordinated actions in order to plan, manage, and complete the requirements of day-to-day procedures or duties. Items were also linked to Doing Housework (n = 27, 1.80%) and Socializing (n = 26, 1.73%). ICF categories with fewer than 20 codes were not detailed for presentation (n = 108, 7.2%).

**Table 2:** ICF content linking analysis: Activities and participation component.

Activities and Participation	ICF Categories and Subcategories	Code Count	Code Percent*
Mobility	d4 (Mobility): 4100 (Lying down), 4102 (Kneeling), 4103 (Sitting), 4104 (Standing), 4105 (Bending),4153 (Maintaining a sitting position), 4154 (Maintaining a standing position) 429 (Changing and maintaining body position), 430 (Lifting and carrying objects), 4451 (Pushing), 450 (Walking), 4500 (Walking short distances), 4501 (Walking long distances), 4551 (Climbing), 4552 (Running), 460 (Moving around in different locations), 4600 (Moving around within the home), 4602 (Moving around outside the home and other buildings), 470 (Using transportation), 475 (Driving)	n = 69	4.59
Self-care	d5 (Self-care): 510 (Washing oneself), 5202 (Caring for hair), 540 (Dressing), 550 (Eating), 560 (Drinking), 570 (Looking after one’s health), 5700 (Ensuring one’s physical comfort), 5701 (Managing diet and fitness), 5702 (Maintaining one’s health)	n = 49	3.26
Remunerative Employment	d850	n = 39	2.59
Carrying out Daily Routine	d230	n = 37	2.46
General Tasks and Demands	d2	n = 30	2.00
Doing Housework	d640	n = 27	1.80
Socializing	d9205	n = 26	1.73
Fewer than 20 codes		n = 108	7.2
TOTAL		n = 385	25.62

*Based on Total Codes (N = 1503)

### ICF code analysis: Environmental factors component

There was little representation of the ICF Environmental Factors component (e codes; n = 11, 0.73%). Immediate Family (e310; n = 3, 0.2%) and Personal Care Providers and Personal Assistants (e340; n = 2, 0.13%) represent the majority of items coded as Environmental Factors. Single items were linked to other Environmental Factors codes, including Assets (e165), Support and Relationships (e3), Friends (e320), Attitudes (e4), and Health Services (e5800).

### Item content analysis

Table 3 summarizes ME/CFS CDEs items based on ICF Component codes. The majority of items included a single meaningful concept linked to Body Functions (b codes; n = 404, 42.80%); a small percent of items linked to Activities and Participation (d codes; n = 70, 7.42%). Only a few items were linked to Environmental Factors (e codes; n = 2, 0.21%). Some items (n = 197, 20.87%) included meaningful concepts for two ICF component codes: Body Functions (b codes) and Activities and Participation (d codes). Multiple items were coded as not definable (n = 41, 4.34%).

**Table 3:** Item content analysis: ICF component.

ICF Codes	Item Examples	Item Count	Item Percent*
Body Functions b codes	I feel weak all over. [Functional Assessment of Chronic Illness Therapy (FACIT)-Fatigue]	n = 404	42.80
Activities and Participation d codes	In the past 30 days, how much difficulty did you have in: Standing for long periods such as 30 minutes? [World Health Organization Disability Assessment Schedule (WHODAS)]	n = 70	7.42
Environmental Factors e codes	Do you live with someone who can take care of you? (Physical Functioning and Activities of Daily Living)	n = 2	0.21
Combined b and d codes	How much did pain interfere with your household chores? (PROMIS Pain Interference)	n = 197	20.87
Not definable	Compared to one year ago, how would you rate your health in general now? [Short Form 36-Item Health Survey (SF-36)]	n = 41	4.34
Items with other code combinations	Other code patterns (e.g., multiple b codes)	n = 230	24.36
TOTAL		n = 944	100

*Percent calculated based on the total number of items (N = 944)

### Fatigue item content analysis

During the linking process, the study team noted that fatigue-related items (n = 251) were often linked to more than one meaningful concept. Table 4 presents meaningful concepts for fatigue-related items. A relatively small percentage of items were linked to single ICF category: Fatiguability (n = 40, 4.24%); Energy and Drive and Energy Level (n = 30, 3.18%). Of the 251 ME/CFS CDEs fatigue-related items, 181 (19.17%) were linked to more than one meaningful concept.

**Table 4:** Item content analysis: Fatigue.

ICF Codes	Item Examples	Item Count	Item Percent*
Fatiguability (b4552)	Physically I feel exhausted. (Checklist for Individual Strength)	n = 40	4.24
Energy and Drive (b130)	I have a lot of plans. (Multidimensional Fatigue Inventory)	n = 30	3.18
Energy Level (b1300)	Mentally tired after the slightest effort. (DePaul Symptom Questionnaire)		
Combined (b130/ b1300 and b4552)	To what degree did you have to force yourself to get up and do things because of your fatigue? (PROMIS Fatigue Short Form)	n = 181	19.17
TOTAL		n = 251	26.59*

*Percent calculated based on the total number of items (N = 944)

Content analysis of three Core ME/CFS CDEs: Core PEM Assessment Questionnaire (S1 Table); DePaul Symptom Questionnaire (S2 Table); and Sleep Questionnaire for All Studies (S3 Table) are presented for review in Supporting Information.

## Discussion

Linking ME/CFS CDEs items to the ICF, an internationally recognized conceptual framework for functioning, disability, and health, provides a foundation to align item content with assessment priorities. Organizing ME/CFS CDEs items by specific ICF content areas can assist clinicians and researchers in selecting CDEs that match intended purposes.

Analysis of ME/CFS CDEs meaningful concepts based on the ICF revealed strong representation of Body Function codes (73.65%) with the highest percentage in ICF categories/subcategories related to the symptom of fatigue. The definition of Fatiguability provides an accurate description of ME/CFS symptoms; however, this ICF subcategory is located under Exercise Tolerance Functions under Functions of the cardiovascular, hematological, immunological and respiratory system, which is not a precise description of fatiguability experienced by persons with ME/CFS. ICF categories Energy Level and Energy and Drive Functions, located under Mental Functions, describe ME/CFS symptoms; however, it is important to note that the ICF does not include a category for energy impairments not related to mental function. Meaningful concepts were also related to other ME/CFS symptoms. ME/CFS CDEs include a wide range of items that assess different aspects of sleep, (i.e., frequency, onset). Persons with ME/CFS report sleep disturbances described as unrefreshing sleep (feeling as tired on waking as when they went to sleep), difficulty falling or staying asleep, reversed sleep cycles and the need for daytime naps [1, 33]. Pain is listed as an “additional common symptom” of ME/CFS by the NAM diagnostic criteria but are not required for diagnosis [1]. ME/CFS CDEs include a relatively high percentage of items assess pain. The majority of CDEs pain items were linked to items assessing Sensations of Pain (b280).

A smaller percent (25.62%) of ME/CFS CDEs meaningful concepts were linked to the ICF Activities and Participation Component. Examination of category/subcategories demonstrates a limited representation of activities that are important to persons with ME/CFS. For example, decreased social interaction is an important participation restriction experienced by persons with ME/CFS [34]; however, only 1.73% of ME/CFS CDEs meaningful concepts pertained to social activities and social interactions. Analysis of content related to Environmental Factors also revealed little representation in ME/CFS CDEs. Examples of the impact of Environmental Factors on the lives of persons with ME/CFS include support from others and accessibility issues. Greater representation of content assessing Environmental Factor*s* can identify participation barriers experienced by persons with ME/CFS.

Analyses to examine the content of ME/CFS CDEs items revealed that a high percentage of items assess a single body function (42.80%) while a small percentage assess a single aspect of activity or participation (7.42%). It is interesting to note that 21% of items assess Body Functions in the context of Activities and Participation. NAM diagnostic criteria emphasize the importance of assessing the impact of symptoms on a person’s ability to engage in everyday activities [1]. PEM, the debilitating hallmark symptom of ME/CFS [35] is a key component of the NAM diagnostic criteria. PEM is characterized by an exacerbation of symptoms and a further reduction in function resulting from a triggering event (e.g., physical, cognitive, sensory, emotional stress). Consequences are often disproportional to the triggering event and effects are prolonged, often lasting several days or longer. Cognitive impairment and orthostatic intolerance, optional components of the NAM diagnostic criteria, may be best assessed in the context of the activities. For example, cognitive impairments present as difficulty performing activities that involve information processing, attention, problem solving, and working memory, as well as other aspects of cognition. Orthostatic intolerance is noted during activities that involve an upright posture (e.g., sitting or standing). ME/CFS assessment items may be improved by applying the ICF framework to develop new items that assess the impact of impairments in Body Function on Activities and Participation.

With the exception of the DePaul Symptom Questionnaire (DSQ2) [36], ME/CFS assessments are generic, or were developed for other conditions [29]. Generic PROMs, developed for the general population, may not include item content with a sufficient range and granularity to assess the symptom severity and extreme activity limitation in the ME/CFS population. A systematic review of PROMs used in ME/CFS noted that few measures involved persons with ME/CFS development, and no studies reported evidence of data quality measurement precision or evidence of measurement responsiveness [29]. However, the DePaul Symptom Questionnaire (DSQ), which was specifically developed to evaluate ME/CFS symptoms and engaged persons with ME/CFS was not included in the systematic review. A study examining the quality of PROs among patients with ME/CFS reported that the DSQ demonstrates reliability, internal consistency and low ceiling effects (<5%) [18].

### Strengths and limitations

A major strength of this work is use of explicit ICF linking protocols [24, 25] to examine ME/CFS CDEs item content. The research team adhered to explicit linking guidelines, implemented an iterative approach, and used two independent coders to mitigate inconsistencies; however, human error is possible.

This work only to applies to the 38 ME/CFS CDEs reviewed as appropriate for administration as PROMs. Limitations of the ICF framework posed several challenges to linking, particularly for concepts related to fatigue and PEM. Previous efforts to define ME/CFS ICF Core Sets [28] identified similar limitations in applying the ICF. Specifically, ME/CFS ICF Core Set developers acknowledged a discrepancy between the clinical manifestations of PEM and available ICF categories; the authors emphasized the need for ICF categories that better describe ME/CFS symptoms [28]. Linking ME/CFS CDEs to energy level in this study should not be construed to indicate that those CDEs describe “mental functions that produce vigor and stamina.” Alternative categories should be considered when further research clarifies the etiology of ME/CFS fatigue- and energy-related symptoms. For example, some items could be linked to metabolic functions that produce energy.

Organization of the ICF Body Function component by systems (e.g., neuromusculoskeletal, movement-related, cardiovascular, hematological, immunological, and respiratory system) does not reflect the fact that ME/CFS is a complex condition that involves multiple body systems. It is important to determine if these ICF categories are valid for describing the fatigue-related symptoms experienced in complex, multisystem conditions, such as ME/CFS. Despite these limitations, the ICF provides an internationally recognized framework that can elucidate constructs assessed by ME/CFS CDEs.

## Conclusion

Study findings build upon the prior credible work to develop ME/CFS CDEs. Many CDE items are relevant for persons with ME/CFS. However, due to the complexity of ME/CFS symptoms, multiple assessments may be required, presenting an unacceptable burden for researchers, clinicians, and persons seeking diagnoses. Application of a conceptual framework to review item content provides a structure for understanding the strengths and limitations of each assessment.

Persons with ME/CFS experience a unique constellation of debilitating symptoms. There is an urgent need to improve assessments for this population. In addition to providing a structure to optimize assessment selection, this review provides a foundation for improving ME/CFS PROMs. Future research is needed to identify optimal structure for PROM item content and address item content gaps. Study findings suggest that focusing on developing items that assess activity limitations and participation restrictions may be an effective strategy to better understand symptom impact. This effort will require qualitative research to develop items that reflect the lived experiences of persons with ME/CFS [37]. Work is currently underway to develop ME/CFS condition-specific PROM item banks. Use of computerized adaptive tests (CATs) to administer these item banks will increase efficiency for researchers and clinicians and decrease patient burden. These item banks will build on current ME/CFS CDEs item content and integrate concepts that emerge from engaging persons with ME/CFS in qualitative research. Analysis of qualitative data will inform item development by illuminating lived experiences, revealing key words and phrases that capture the unique lexicon of descriptors used by persons with ME/CFS.

This work is particularly relevant and timely given the similarities between the symptoms of ME/CFS and Post-acute sequelae of SARS-CoV-2 infection (PASC) [38–43], highlighting the importance of identifying strategies to improve assessments for these complex conditions. Using a well-accepted conceptual framework to examine ME/CFS CDEs item content is an important first step to select targeted and streamlined assessments.

## Supporting information

S1 Table Content analysis: Core PEM Assessment Questionnaire. (PDF)

S2 Table Content analysis: DePaul Symptom Questionnaire. (PDF)

S3 Table Content analysis: Sleep Questionnaire for All Studies. (PDF)

S1 Appendix ME/CFS CDE assessments excluded from analyses. (DOCX)
