# Supplementary material for: Myalgic Encephalomyelitis—Chronic Fatigue Syndrome Common Data Element item content analysis
Source: PLoS One. 2023 Sep 12;18(9):e0291364. doi: 10.1371/journal.pone.0291364 (PMC10497138; doi:10.1371/journal.pone.0291364)
Supplement: S1 Table — (PDF) [file pone.0291364.s001.pdf]

S 1 Table. Core PEM Assessment Questionnaire (5 items)

Unique ICF codes = 4

Level 1(n=0)	Level 2 (n=0)	Level 3 (n=10)	Level 4 (n=0)
		b1300 Energy level (n=1)	
		b455 Exercise tolerance functions (n=1)	
		b4550 Physical endurance (n=3)	
		b4552 Fatiguability (n=5)	
