# Supplementary material for: Myalgic Encephalomyelitis—Chronic Fatigue Syndrome Common Data Element item content analysis
Source: PLoS One. 2023 Sep 12;18(9):e0291364. doi: 10.1371/journal.pone.0291364 (PMC10497138; doi:10.1371/journal.pone.0291364)
Supplement: S2 Table — (PDF) [file pone.0291364.s002.pdf]

S 2 Table. DePaul Symptom Questionnaire (157 items)
Unique ICF codes = 61

Level 1(n=8)	Level 2 (n=52)	Level 3 (n=134)	Level 4 (n=12)
b2 Sensory functions and pain (n=2)	b134 Sleep functions (n=1)	b1300 Energy level (n=31)	b21020 Light sensitivity (n=2)
d2 General tasks and demands (n=4)	b140 Attention functions (n=2)	b1302 Appetite (n=2)	b28010 Pain in head and neck (n=4)
d7 Interpersonal interactions and relationship (n=1)	b144 Memory functions (n=4)	b1341 Onset of sleep (n=2)	b28011 Pain in chest (n=2)
d8 Major Life areas (n=1)	b152 Emotional functions (n=1)	b1342 Maintenance of sleep (n=4)	b28012 Pain in stomach or abdomen (n=2)
	b164 Higher-level cognitive functions (n=2)	b1343 Quality of sleep (n=6)	b28016 Pain in joints (n=2)
	b210 Seeing functions (n=1)	b1400 Sustaining attention (n=2)	
	b230 Hearing functions (n=2)	b1402 Dividing attention (n=1)	
	b280 Sensation of pain (n=2)	b1443 Working memory (n=2)	
	b435 Immunological system functions (n=3)	b1561 Visual perception (n=1)	
	b440 Respiration functions (n=2)	b1600 Pace of thought (n=2)	
	b455 Exercise tolerance functions (n=2)	b2100 Visual acuity functions (n=2)	
	b515 Digestive functions (n=1)	b2401 Dizziness (n=2)	
	b530 Weight maintenance functions (n=2)	b2402 Sensation of falling (n=2)	
	b535 Sensations associated with the digestive system (n=1)	b2700 Sensitivity to temperature (n=1)	
	b550 Body temperature (n=2)	b2703 Sensitivity to noxious stimulus (n=2)	

	b620 Urination functions (n=1)	b2800 Generalized pain (n=2)	
	b765 Involuntary movement functions (n=2)	b2801 Pain in body part (n=2)	
	b830 Other functions of the skin (n=4)	b4101 Heart rhythm (n=2)	
	d310 Communicating with receiving spoken messages (n=1)	b4353 Functions of lymph nodes (n=2)	
	d570 Looking after one's health (n=5)	b4550 General physical endurance (n=4)	
	d640 Doing housework (n=2)	b4552 Fatiguability (n=35)	
	d760 Family relationships (n=2)	b5135 Tolerance to food (n=2)	
	d850 Remunerative employment (n=2)	b5350 Sensation of nausea (n=2)	
	d920 Recreation and leisure (n=5)	b5351 Feeling bloated (n=2)	
		b5508 Thermoregulatory functions (n=12)	
		b7206 Power of all muscles of the body (n=2)	
		b7650 Involuntary contractions of muscles (n=1)	
		d5702 Maintaining one's health (n=3)	
		d9205 Socializing (n=1)	
