# Supplementary material for: Myalgic Encephalomyelitis—Chronic Fatigue Syndrome Common Data Element item content analysis
Source: PLoS One. 2023 Sep 12;18(9):e0291364. doi: 10.1371/journal.pone.0291364 (PMC10497138; doi:10.1371/journal.pone.0291364)
Supplement: S3 Table — (PDF) [file pone.0291364.s003.pdf]

S3 Table. Sleep Questionnaire for All Studies (16 Items)

Unique ICF codes = 6

Level (n=0)	Level 2 (n=2)	Level 3 (n=22)	Level 4 (n=0)
	b134 Sleep functions (n=2)	b1300 Energy level (n=2)	
		b1341 Onset of sleep (n=3)	
		b1342 Maintenance of sleep (n=6)	
		b1343 Quality of sleep (n=9)	
		b4552 Fatiguability (n=2)	
