# Supplementary material for: Myalgic Encephalomyelitis—Chronic Fatigue Syndrome Common Data Element item content analysis
Source: PLoS One. 2023 Sep 12;18(9):e0291364. doi: 10.1371/journal.pone.0291364 (PMC10497138; doi:10.1371/journal.pone.0291364)
Supplement: S1 Appendix — (DOCX) [file pone.0291364.s004.docx]

Appendix 1: ME/CFS CDEs Not Included in Analysis

1. Adolescent Sleep Hygiene Scale (ASHS)
2. Adult Employment and Education History
3. Automated Neuropsychological Assessment Metrics (ANAM)
4. Beighton Score
5. Biomarker-Related Sample and Collection Questions
6. Biomarkers Guidelines
7. Biomarkers Reference Table
8. California Verbal Learning Test - Children (CVLT-C)
9. California Verbal Learning Test - Second Edition (CVLT-II)
10. Cambridge Neuropsychological Test Automated Battery (CANTAB)
11. Cardiopulmonary Exercise Testing (CPET)
12. Child Health Questionnaire
13. Children's Memory Scale (CMS)
14. Children's Paced Auditory Serial Addition Task (ChiPASAT)
15. Cogstate Tests
16. Deary-Liewald Reaction Time Task
17. Delis-Kaplan Executive Function System (D-KEFS)
18. Demographics
19. Diffusion Tensor Imaging (DTI)
20. DSQ-Pediatric Screening Questionnaire (DSQ-PSQ)
21. Edinburgh Handedness Inventory
22. Epworth Sleepiness Scale - Children's Version
23. Exposure History
24. Faces Pain Scale - Revised (FPS-R)
25. Family Health History
26. Fatigue Visual Analog Scale
27. Fatigue/Activity Record and Diary
28. Functional Disability Inventory-Child and Adolescent Form (FDI)
29. Functional Disability Inventory-Parent Form (FDI)
30. Functional Magnetic Resonance Imaging (fMRI),
31. General Core
32. Grip Strength Fatigue
33. Guidance for Core PEM Assessment
34. Guidance for PEM-Focused Studies
35. Kiddie-Schedule for Affective Disorders and Schizophrenia for School-Aged Children-Present and Lifetime Version (K-SADS-PL)
36. Laboratory Test Results
37. Laboratory Tests - Immune Module
38. Low Resolution Electromagnetic Tomography (LORETA)
39. Magnetic Resonance Imaging (MRI)
40. Magnetic Resonance Spectroscopy (MRS)
41. Magnetoencephalography (MEG)
42. ME/CFS Functional Imaging Task Summaries
43. ME/CFS Symptom Checklist
44. Medical History - Immune Module
45. Medications/Other Treatments
46. Modifiable Activity Questionnaire (MAQ)
47. Multidimensional Fatigue Scale
48. Neuroendocrine Labs
49. Neuroendocrine/Hypothalamic Symptoms
50. Neurological Exam
51. NIH Toolbox Cognitive Battery
52. Non-Quantitative Electroencephalography (EEG)
53. North American Adult Reading Test (NAART)
54. Paced Auditory Serial Addition Test (PASAT)
55. Pain Assessment
56. Pain Frequency-Severity-Duration Scale (PFSD)
57. Passive Standing Test Protocol
58. Past and Current Illnesses
59. Pediatric Quality of Life Inventory
60. PEM-Focused Studies Questionnaire
61. Physical Exam - Immune Module
62. Physical Examination
63. Polysomnography (PSG) for ME/CFS
64. Positron Emission Tomography (PET)
65. Quantitative Electroencephalography (qEEG)
66. Reproductive and Hormonal History
67. Rey-Osterrieth Complex Figure Test (ROCF)
68. Sleep Focused Study Questionnaire
69. Special Imaging Considerations
70. Speeded Tapping Test
71. Stanford Sleepiness Scale
72. Stroop Test
73. Suggested Parameters for Common Magnetic Resonance Imaging (MRI) Scans
74. Test of Premorbid Functioning (TOPF)
75. Test of Variables of Attention (TOVA)
76. Wechsler Abbreviated Scale of Intelligence - Second Edition (WASI-II)
77. Wechsler Adult Intelligence Scale - Fourth Edition (WAIS-IV)
78. Wechsler Intelligence Scale for Children-Fifth Edition (WISC-V)
79. Wechsler Memory Scale IV (WMS-IV)
80. Woodcock-Johnson III Test of Cognitive Abilities
81. Word Reading Subtest of the Wide Range Achievement Test (WRAT-4)
